# The Right to A Clean Environment: Considering Green Logistics and Sustainable Tourism

**DOI:** 10.3390/ijerph17093254

**Published:** 2020-05-07

**Authors:** Dalia Perkumienė, Rasa Pranskūnienė, Milita Vienažindienė, Jurgita Grigienė

**Affiliations:** 1 Business and Rural Development Research Institute, Faculty of Bioeconomy Development, Agriculture Academy, Vytautas Magnus University, Kaunas 53361, Lithuania; rasa.pranskuniene@vdu.lt (R.P.); milita.vienazindiene@vdu.lt (M.V.); 2 Faculty of Law, Vytautas Magnus University, Kaunas 53361, Lithuania; jurgita.grigiene@cerka.lt

**Keywords:** right to a clean environment, sustainable tourism, green logistics solutions, climate change

## Abstract

The globalization process has yielded various undesirable consequences for the environment and society, including increased environmental pollution, climate change and the exhaustion and destruction of resources. The influence of these processes makes it difficult to guarantee citizens’ rights to a clean environment, and the implementation of this right requires complex solutions. The aim of this integrative review article is to discuss the right to a clean environment, as it relates to green logistics and sustainable tourism, by analyzing various scientific and legal sources. Rethinking the possible solutions of green logistics for sustainable tourism, such as tourism mobilities, bicycle tourism, the co-creation of smart velomobility, walkability, and others, can help us also rethink how to balance, respect, protect, and enforce human rights in the present-day context of climate change challenges. The integrative review analysis shows the importance of seeking a balance between the context (the right to a clean environment), the challenge (climate change), and the solutions (green logistics solutions for sustainable tourism).

## 1. Introduction

The rapid globalization processes in the natural environment and society cause various undesirable consequences, such as increased environmental pollution, climate change, and the exhaustion and destruction of natural resources. These processes degrade the quality of life, endanger human health (and even life), and limit and violate the social rights of individuals to live in a clean and healthy natural environment. The influence of these processes makes it very difficult to guarantee citizens’ rights to a clean environment. Protecting the environment and ensuring people’s rights to a clean and healthy environment is one of the priority areas of law, economics, and politics. The concept of human right to a clean, high quality, and healthy natural environment, the relevant legal regulations, and the implementation of those rights have been studied by various scholars [1,2,3,4,5,6,7,8,9,10]. 

Tourism, as one of the fastest growing industries [11,12], is one of the major contributors to the greenhouse effect and CO_2_ emissions [13]. Climate change, and its potentially substantial impacts on tourism worldwide, is a pressing issue [14]. Leisure travel significantly increases CO_2_ emissions due to the transportation used during people’s holidays [15]. Lenzen et al. [13] showed that the pursuit of economic growth comes with a significant carbon burden, as tourism is significantly more carbon-intensive than other potential areas of economic development. Fang et al. [16] noted that the interaction between climate change and tourism has been one of the most critical and dynamic research areas in the field of sustainable tourism in recent years. As the current situation is untenable, so in the present, the concept of sustainability is one of the key issues worldwide [17]. The tourism sector is no exception and contributes to the challenges of sustainability. Moreover, the sustainability conversion of the tourism sector, both globally and socially, determines not only legal and political structures but also societal provisions and individual values [18]. The scientific sources related to sustainable tourism and its development present various research positions. Part of them [11,19,20,21] focus on examining the concept of sustainable tourism, its importance, and the appropriate methods to achieve it. Other academics have studied green hotels [22,23,24,25], discussed the economic development of the tourism industry [26,27,28], debated the collaboration and partnership development of sustainable tourism [12,29,30]. Based on the opinions of these researchers, tourism can be sustainable if there is close cooperation and sharing between various industries, as well as between tourists, hotels, communities, and public authorities. Various researchers have studied tourism [31,32,33] and climate change [34,35,36,37,38,39,40,41,42,43]. These authors noted that without major changes in thinking and purposeful activities on sustainable tourism, the development of tourism will be impossible. 

Sustainable tourism development also requires the application of green activities and innovations. Various authors [44,45,46,47,48,49] noted that one of the challenges for the development of sustainable tourism is the implementation of the principles and initiatives of green solutions. Green solutions and sustainable tourism are very closely linked, especially in the use of green logistics and its activities, such as the management of tourist flow (especially in resort areas), reducing traffic flow and noise levels, and other environmental factors. These sectors are closely linked through laws. The concepts of green logistics and green transport have been analyzed from different perspectives. Nilnoppakun [50] and Lin et al. [51] addressed the issue of green logistics and green transport development for tourist attractions and suggested an initiative for the superior development of green transport from a government and enterprise perspective. Researchers analyzed various greening processes and solutions in the tourism sector [52], emphasized the main tasks of green transport in the area of reducing the negative environmental impacts of households and entities operating in the city [53]. Khan et al. [54] underlined that application of green transport practices as the only solution to control air pollution, climate change, and global warming problems. The studies about drone food delivery services as green solutions, having the great potential to save the environment, have emerged in recent years [55,56,57,58]. Hanna et al. [59] debated the advocacy for sustainable transport, and Oklevik et al. [12] presented ideas about the effects of perceived traffic risks, noise, and exhaust on bicyclist behavior. However, there are studies related to the innovations of green logistics in the context of sustainable tourism. Pan et al. [60] provided an overview of the interrelationships between tourism and sustainability from a cross-disciplinary perspective and underlined the main elements of sustainable tourism, such as green energy, green transportation, green buildings, green infrastructure, green agriculture, and smart technologies, which should be linked to green logistics initiatives.

As sustainable tourism and green logistics are broad and complex fields, green logistics is discussed in this study to seek out innovative ideas, exploring how green logistics solutions can contribute to the challenges of sustainable tourism development and how they influence decisions while helping to achieve the right to a clean environment. Climate change also is discussed as one of the main challenges to achieving the right to a clean environment in the context of sustainable tourism. Thus, the aim of this integrative review article is to discuss the right to a clean environment through green logistics and sustainable tourism by analyzing various scientific and legal sources. 

This work is organized as follows. Subsequent sections present the materials and methods, results, discussion, and concluding insights. The literature review method is presented in the materials and methods section. The results are discussed in the results chapter (the review data are organized under the following themes: the right to a clean environment and its legal regulation; tourism challenges: between climate change and sustainability; and green logistics solutions for sustainable tourism. The discussion section debates how balance should be sought for the right to a clean environment. This integrative review finishes by offering concluding insights.

## 2. Materials and Methods

Literature reviews, as Torraco [61] notes, are conducted differently for various audiences and for different purposes. This integrative review paper seeks to undertake an interdisciplinary discussion about the right to a clean environment considering green logistics and sustainable tourism. For this paper, the integrative review approach outlined by Whittemore and Knafl [62] was adopted: “The integrative review method is an approach that allows for the inclusion of diverse methodologies, and contributes to the presentation of varied perspectives on a phenomenon of concern”. Additionally, Whittemore and Knafl [62] noted that integrative reviews are the broadest type of research review method that allows for the simultaneous inclusion of experimental and non-experimental research in order to more fully understand the phenomenon being studied. An integrative review may also combine data from theoretical as well as empirical literature [62]. The aim of this integrative review is not only to analyze general laws and scientific opinions but also to interpret the various aspects that are associated with the right to a clean environment, as well as those related to sustainable tourism and green logistics. We have chosen to examine the special tensions related to the right to a clean environment, so the selected international legal documents and cases focus on issues in this field.

We use five stages for this review [62]: identification of the problem, a search of the literature, evaluation of the data, analysis of the data, and presentation (Table 1).

## 3. Results

As mentioned for the integrative review, we examined articles discussing legal documents related to a clean environment (Table 2); tourism, climate change, and sustainability (Table 3); and green logistics solutions for tourism (Table 4). The integrated review data were organized under the following themes: the right to a clean environment and legal regulations (3.1); tourism challenges considering climate change and sustainability (3.2); and green logistics for sustainable tourism (3.3).

### 3.1. The Right to a Clean Environment and Its Legal Regulations

The right to a clean environment is divided into two parts: First, the concept of a clean environment is discussed, and then the legal regulations for a clean environment are presented more deeply. The legal documents and cases selected for the analysis are presented in Table 1. As seen in Table 1, the cases of the review run from 1948 to 2016, which ensures a selection of classical and typical cases related to this theme. 

#### 3.1.1. The Right to a Clean Environment

When analyzing the right to a clean environment, the concept of the environment should be defined first. Thorme [2] defines the environment as “a human right” and clearly emphasizes that this entails the right to a “safe, healthy, and environmentally sustainable environment” [2]. However, a deep and comprehensive analysis of the regulations of these rights at the international and EU levels are not very common [3,8]. Moreover, most countries’ national constitutions use similar concepts of a clean environment. For example, the Portuguese and Lithuanian Constitutions speak about the constitutional right to a clean environment [63,64]. The meaning of “a clean environment” here is ambiguous as the term may refer to freedom from crime or the threat of pollution. For example, children have the right to a safe environment at school [65]. Lewis [9] discusses the nature of the relationship between the environment and human rights in international law and recognizes that this relationship is undisputed. The author distinguishes between the concept of a human right and a good environment. Lewis [9] sees climate change as the greatest environmental challenge and examines whether the right to a good environment can provide new approaches to the human rights implications of climate change (see Ashgar Leghari v. Federation of Pakistan case [66]). Analyzing the concept of a clean environment, lawyers and scholars have explored the relationship between human rights and the right to a clean environment from different perspectives. Some authors, such as Fitzmaurice [67] and Lansdown et al. [68], highlight children’s rights to a clean and safe environment. Shelton [4] identifies three main strands of human rights to a clean environment: 1. The absence of the right to a clean environment; 2. The existence of the right to a clean environment; and 3. The right to a clean environment stemming from other inherent human rights (e.g., the right to life, health, and information) [3,4,9]. Dogaru [6] argues that the right to a clean and safe environment is a fundamental human right, the nature and characteristics of which do not change over time. In the scientific legal literature, the right to a clean environment is associated with others human social, economic, cultural, political, and civil rights [5]. Fundamental human rights are inalienable, and this principle applies to the right to a healthy and a safe environment [1,5]. 

Thus, the right to a clean environment, understood as the relationship between the environment and humans, should help regulate individual rights and the opportunity to live in a clean and safe environment. The individual’s right to a clean environment is a fundamental human right that derives from other inherent human rights. Table 2 presents a description of the literature on relevant legal cases and conventions.

#### 3.1.2. Legal Regulation of the Right to a Clean Environment

The right to a clean and safe natural environment is also enshrined in the legal instruments of the European Union. The rights of persons to a clean and safe environment are also enshrined in the Universal Declaration of Human Rights [78]. This declaration emphasizes the rights of individuals to a safe, healthy, and clean environment. The Stockholm Declaration on the Human Environment [79] also declared the human right to a safe and clean environment. Another important declaration is the Rio Declaration on the Environment and Development [80], which emphasizes the human right to “a healthy and full life in harmony with nature”. The Rio Declaration is closely related with the Stockholm Declaration and is a key piece of environmental legislation. The Rio Declaration emphasizes that people have the right to a healthy and a productive life in harmony with nature [10]. The human right to a clean and a healthy environment is also highlighted in the Treaty of Maastricht, which amended the treaties establishing individual communities to establish the European Union. In the preamble to the EU Treaty, the Member States are said to “promote the economic and the social development of their peoples, with a view to promoting the greater cohesion and the protection of the environment” [81]. It is also important to mention the Kyoto Protocol, which aims to reduce the greenhouse effect by limiting CO_2_ emissions [82]. People’s right to a safe environment is also closely related to the Treaty of Amsterdam, whose preamble entrenches the principle of endless development and stronger protection of the environment [83]. The importance of the Aarhus Convention [84] for ensuring a clean and safe environment should also be noted in the analysis of the international environmental instruments. In order to protect the fundamental rights of individuals, such as the right to a healthy, safe, and clean environment, the European Court of Justice often emphasizes the international treaties of Member States related to the protection of these rights. The European Convention on Human Rights is the main treaty on this subject. Moreover, Article 6 (1) of the Maastricht Treaty imposes an obligation on the European Union to respect the fundamental rights of each Member State [81]. Compliance with and enforcement of a contract can be upheld as a court decision, as in the case of the Nold v. KG commission [69], which upheld these rights. The case law of the European Court of Human Rights addresses issues related to the right of each individual to a healthy, safe, and clean environment with respect to the right to life, the right to private and family life, freedom of expression, etc. Cases of pollution, noise, odors, etc., which may affect people’s well-being or pose a direct threat to health, have also been examined [85]. The European Convention for the Protection of Human Rights and Fundamental Freedoms does not directly enshrine the right to a healthy, safe, and clean environment. However, the European Court of Human Rights (ECHR) links this right with the right to life, the right to a private and family life, freedom of expression, and others. The interpretation of this legal provision in the context of environmental protection is found in the ECHR judgment of Öneryıldız v. Turkey [70]. Above all, this obligation arises in relation to dangerous activities, whether carried out by a private or public entity [70]. International courts often deal with cases involving the greenhouse effect and emissions, particularly CO_2_ [67]. In the case Fadeyeva v. Russia [72], the ECHR noted that, in order for complaints concerning environmental factors to fall within the scope of Article 8, environmental factors must first be proven to actually affect the scope of the applicant’s private life, as well as the degree of severity of the exposure, which is determined by the intensity, duration, physical, and mental impacts of the adverse effects, as well as the overall environmental context. Similar violations of an individual’s right to life as the result of the harmful effects of the environment and the right to a private and family life have also been analyzed in other cases, such as Budayeva et al. v. Russia [73], the ECHR case X v. Iceland 5 DR 86 [74]; Kelsey Cascade Rose Juliana et al. v the United States of America, Tatar v. Romania [76], etc. In the ECHR case Kolyadenko v Russia [77], 56 EHRR 2, the applicants alleged that the State was responsible for putting their lives at the risk and for the damage done to their homes and property as a result of the sudden large-scale evacuation of water from the Pionerskoye reservoir and the ensuing flooding in the area around the reservoir. The applicants also complained that they had not received effective remediation in the matter.

In conclusion, legal instruments aim to maximize the assurance of the implementation of citizens’ rights by addressing climate change and other environmental challenges. This area remains problematic, especially in international law. In this situation, the question is whether the implementation of legal norms could be enhanced between different areas such as tourism and green logistics. What are the possible solutions? Therefore, the next section of this article discusses tourism challenges in the context of climate change and sustainability. 

### 3.2. Tourism Challenges: Concerning Climate Change and Sustainability

The tourism sector bears a large responsibility for the quality of the environment to ensure the fulfillment of an individual’s right to a clean environment. The relationship between tourism and environmental quality assurance is complicated [86] and covers a range of activities that may have a negative impact on the environment. Many of these tourist activities that have a negative impact on the environment are related, for example, to roads and the airports, as well as to tourist attractions [21]. The negative impacts of hotels, marinas, restaurants, and shops on the environment should also be mentioned. The negative impacts of tourism development are detrimental to the environment and environmental resources. Muler Gonzalez et al. [87] pointed out that the negative impact of tourism is also particularly visible under an increase in tourist flow in the case of overtourism, which can lead to undesirable and environmentally harmful effects, such as increased pollution, soil erosion, loss of natural habitats, and increased forest fires. Traveling for leisure purposes is not a necessity, but it contributes significantly to CO_2_ emissions [13]. Tourism also includes the transportation of tourists to and from their accommodations. It is estimated that transport accounts for about 75% of tourism’s CO_2_ emissions and aviation counts for about 40% [88]. The improvements in energy efficiency in the transport sector are expected to lead to an increase in energy efficiency between 2005 and 2035. This will reduce the emissions per passenger kilometer by up to 32% [15].

Moreover, tourism should be sustainable to achieve the individual right to a clean environment. Sustainable tourism is not an isolated or special form of tourism; rather, all types of tourism should become more sustainable. Thus, increasing tourism in a more sustainable way does not only mean controlling and managing the negative effects of the industry. Economic development and environmental protection should not be seen as opposing forces but should instead be pursued together, as sustainable tourism practices can only be attained when the holistic principle of sustainability is understood and integrated into the strategic planning of the industry [20,89]. Sustainable tourism is often also referred to as responsible tourism [90], which has been adopted by industries who feel that the word sustainability is overused. A challenging issue here is ecotourism, which refers to responsible travel to natural areas that preserve the environment. The idea of ecotourism, according to Krüger [91], is a form of nature-based tourism, contributing both towards socioeconomic and environmental benefits. This concept entered the scientific, and later public, consciousness in the 1990s and presently faces challenges due to its popularity. Voumard [92] noted that eco-tourism theoretically consists of responsible travel to natural areas, which benefits both environment and social goals. Ignoring these positive goals, Voumard highlighted the uneven results of eco-tourism development, singling out the gaps between the promised and observed results. Although eco-tourism yields economic benefits, it can also have unintended negative consequences for the conservation of wildlife in protected areas [93]. The challenging issue of seeking sustainability in ecotourism led us to examine one more aspect of sustainable tourism. Responsible tourism seeks to minimize the negative social, economic, and environmental impacts and to ensure citizens’ rights to a clean and safe environment. Responsible tourism is about making “better places for people to live in better places for people to visit” [94]. We argue that one of the key factors for a good place to live and also a place to visit is a safe and clean environment. Therefore, sustainable tourism as responsible tourism seeks to ensure and enforce people’s rights to a clean environment. This relationship is a result of the close cooperation between law and tourism. This cooperation remains very challenging and debatable. Table 3 presents a description of the literature on tourism, climate change, and sustainability.

Notably, tourism is increasingly affected by the same effects of climate change: Some areas are expected to become too hot for the tourists, while other coastal areas may be flooded [95,96]. Hall et al. [40] noted that tourism is an extremely significant global activity. Tourism is also a major global phenomenon, as Mearns [38] argues, which is highly sensitive to climate change but is, at the same time, a major contributor to climate change that could have significant impacts on natural and manmade attractions worldwide.

It is difficult today to imagine a world without tourism [38]. In order to be properly understood, the phenomenon of tourism should be clearly and comprehensively defined to assess its true meaning and purpose. The development of the tourism industry, as Wang et al. [28] noted, caused many environmental problems, but, the implementation of low-carbon technologies has recently become an inevitable choice for tourism development. Moreover, the rapid development of the tourism industry has caused many losses, which requires the introduction of low-carbon technologies in order to achieve sustainable development. According to Scott et al. [37], there is very little knowledge on the abilities of current tourism operators and communities to adapt to potential changes related to climate change issues, as there is a growing lack of confidence in the current interpretations of the real impact of climate change on the tourism industry. Mearns [38] observes that with the tourism industry drastically reducing its contribution to climate change, all long-distance travel should be stopped in order to limit climate impacts. On the other hand, many poor countries across the world are heavily dependent on these long-distance travelers to create jobs and reduce poverty [96,99]. The thriving local communities, as Mearns [38] argues, living in intact natural landscapes are critical for tourism’s long-term sustainability and viability. Juvan and Dolnicar [100] discussed the pro-environmental behavior of tourists, noting that during holidays, the treatment of the environment decreases during all types of holidays; de Bruijn et al. [101] also noted that the carbon footprint of travelers doubles when they go on holiday. Baldner [102] noted that such pro-environment attitudes may be driven by a desire to protect individuals while protecting the environment, while an anti-environmental approach may be driven by a desire to maintain the status quo. Traveling to holiday destinations should not cost us the Earth; instead, it should create better places for people to live in and for people to visit. In tourism policy terms, Hall et al. [41] note that sustainability is primarily seen as being ‘environmental’ and development as ‘economic’ (and to a lesser extent, ‘social’); the concept of sustainable tourism, or sustainable tourism development, seeks to mitigate the paradox between these two understandings without fundamentally affecting existing economic relationships [103]. Continuing growth in aviation and tourism emissions are clearly in conflict with global climate change and greenhouse gas (GHG) reduction goals [41]. Despite enthusiasm for changing consumer tourism behavior, people seem to be reluctant to give up their international travel or to make other important changes [37,99]. 

Overall, the role of the tourism sector should help to ensure the quality of the environment. Could tourism enforce a citizen’s right to a clean environment? Grimm et al. [39] suggest that the entire tourism system can and should take steps to reduce the GHG emissions associated with its activities. It seems important to note that green solutions in logistics (another challenging sector) could also contribute to the maintenance of a clean environment, so the next section of this article discusses the challenges and possible green solutions that could contribute fulfilling the right to a clean environment.

### 3.3. Green Logistics Solutions for Sustainable Tourism

Due to the impact of green logistics as possible green solutions for climate change, as well as increasing air pollution, noise, and traffic accidents, green logistics has recently been receiving increasingly more attention. In academia, debates on green logistics are often associated with the concept of sustainable development. Green logistics can be defined as all attempts to minimize the ecological impact of logistics activities [104]. Further, green logistics is a research field that aims to assess and reduce the environmental impact of logistics [105]. Logistics systems are created in accordance with human needs and interests and reflect the trends in strategies for implementing sustainable development [44,48]. Scott et al. [106] observed that the term “green logistics“ is often used interchangeably with “reverse logistics“, but in contrast to reverse logistics, green logistics “summarizes the logistics activities that are primarily motivated by the environmental considerations” [107]. As green logistics and sustainable tourism concepts are closely intertwined, complementing each other to preserve a high quality and clean environment, all efforts in the green logistics area are, therefore, focused on contributing to sustainability [108]. Could green logistics solutions for more sustainable tourism facilitate a better implementation of citizens’ rights to a safe and clean environment?

Seroka-Stolka et al. [48], Gavrilović et al. [52], Pan et al. [60], and Wang et al. [109] emphasized that the implementation of green logistics initiatives for sustainable tourism should be based on the economic, ecological, and social responsibility levels that are equivalent and complementary. According to Broman and Robèrt [110], ecological, social, and financial capital is essential for sustainable society and for the transition to a such society. The clear link between logistics and tourism can be seen in green transportation, which has a direct impact on the efficiency of sustainable tourism, as well as fuel-efficient vehicles, biofuels, electric vehicles, bicycles, and tricycles for delivery. Thus, governmental authorities should enforce green practices in logistics and transport-related operations, to increase tourist safety and security, which may mitigate adverse effects on environmental sustainability and also attract tourists [111].

Peeters et al. [97] focused on the challenge of mitigating climate change, which is critical to the future of desirable tourism transportation. However, to date, relatively little attention has been paid to this aspect of sustainable tourism. Smith et al. [112] noted that tourism policy directions have evolved over several years and in different contexts for many tourism destinations and that it is far from clear what a desirable transport future looks like. Thus, what could be the possible solutions here? The possible solutions for green logistics for sustainable tourism, such as mobility tourism, bicycle tourism, and the co-creation of smart velomobility and walkability are discussed in the next section. A description of the literature on green logistics solutions for tourism is shown in Table 4. 

#### 3.3.1. Tourism Mobilities

In recent years, increasingly more research about tourism mobilities has emerged. Hanna and Adams [59] analyzed the ways in which participants understand and apply the potential conflict between the concept of sustainable holidays and air transport. Kantenbacher et al. [115] presented research findings suggesting that voluntary reductions in flying are more likely than other forms of environmentally friendly sacrifice. As Scuttari et al. [116] point out, in ecologically sensitive but intensive tourism locations, transport policy-makers face the paradox of realizing the need to minimize transport-related impacts but failing to reverse the current situation. The authors noted that this situation occurs because the recognized negative impacts of traffic on the tourism economy are understood as less problematic than the potential impacts of traffic management. The authors [108] noted that alternative transportation is expected to be efficient, frequent, cheap, and integrated with a long daily schedule and that restrictions on private mobility should be clearly laid out. Tourism is in a state of the change, as Nilsson [21] points out, especially in urban areas where innovations such as low-cost aviation and digital reservation platforms have created new dynamics, including new models for tourist activities and mobility spaces. In trying to mitigate the negative impacts of urban tourism, which are caused by tourism transport, Nilsson [21] observed that the efforts to promote participation in non-motorized transport have gradually become particularly relevant and interesting. Through an in-depth life-history approach, Cass and Faulconbridge [122] contributed to the understanding of mobility focused on the emotional experience of mobility. According to these authors, rethinking ideas about the value of travel time, which focuses on mobility, can be as effective as the time used to engage in other practices. They also emphasize that this factor is important because it reveals the tools for creating mobility cultures and meaningful mobility performance. Recent research shows the importance of discussing tourism mobilities. Drones, as one more challenging solution, emerging in green research, would seem having the great potential to save the environment [55,57], especially in drone food delivery services, while debates about passenger drones [113] on the topic of environment and sustainability are mostly characterized by a lack of solid scientific evidence and uncertainties about the environmental impact. Hoed [114] also provides empirical evidence for the newer concepts of tourism mobility closer to the structure of everyday life while more explicitly recognizing that tourism mobility occurs in, rather than away from, the space and time of daily commitments. In addition, it emphasizes the wide variety of tourism practices that are carried out indirectly or directly as part of a mobile life. Docherty [127] notes that there will undoubtedly be a shift towards a more advanced future for mobility, which will have a major impact on the role of mobility in society. 

#### 3.3.2. Green Tourism Transport Solution—Bicycle Tourism

Nilsson [21] positively describes urban cycling tourism development as a state-of-the-art technological process and describes the changes in regional social and technological mobility. Based on his many years of experience, Lee [125] observes that the participants in relevant research can break free from their unconscious car models and become the embodied bicycle carriers who enter into an active negotiation with their commuting practices. A holistic approach is needed for a better understanding of tourism’s role. Nilsson [21] suggests that, in this sense, cities can be considered as creative areas of innovation where relative innovations, such as urban cycling tourism, can be of interest to urban tourism in general. Cycling is considered an authentic local experience and can become a part of the tourism location. Cycling, as Hoed [114] observes, is an affordable mode of transport that provides an opportunity for older people to recover and socialize. In many countries, older people rarely cycle and are mostly dependent on motorized travel. The author [109] also observes that, given a slower approach to tourism, cycling provides a lively experience of (tourism) mobility as an object for analysis and assumes the role of non-motorized travel. Kingham and Tranter [128] noted that cycling is environmentally, socially, and economically sustainable, and Wang and Wen [109] emphasized that cycling can be a viable alternative to private cars for short trips and can be more effective than walking for improving one’s health because it is more intensive. According to Zhang et al. [111], bicycle sharing is a good example of environmentally friendly traveling and an innovative solution to meet people’s future needs for mobility in urban environments. Nilsson [21] also emphasizes that there are several types of cycling tourists. The predominant type in the literature is fit cycling tourists [21]. For this type of tourist, cycling is an important element of travel and is used for sports, long trips, or to organize several excursions. Such cycling tourists usually visit rural areas. Another, less visible, category involves holiday cyclists. For these tourists, cycling is a part of their holiday experience but is not their primary focus [129]. Such cyclists, due to their narrow definition, cannot be considered suitable bicycle tourists [130], but they are nonetheless interesting as an element of urban tourism [21]. They, as Nilsson [21] observes, take tours or are a day-trippers who use bicycles in more commonplace ways than special-interest bicycle tourists. 

#### 3.3.3. Green Tourism Transport Solution as a Co-Creation of Smart Velomobility

Berrada [123] observes that the concept of co-creation in tourism is underdeveloped in the literature and that the researchers in this exciting field continue to place the tourist down the chain, limiting tourists to a responsive role in the tourism experience designed and delivered by one or more providers. In the logic of tourism value creation, tourism companies must treat tourists as participants in product development and tourism experiences, not just as spectators [118]. The concept of velomobility relates to mobility research related to cycling. Behrendt [124] notes that smart velomobility considers how physical and digital spaces are created around and during cycling, as well as how velocity data is created, shared, and analyzed in individual and collective/fleet use. As the author observes, this concept relates to physical mobility, infrastructure, power relationships, representations, and everyday experiences and practices by exploring the digital and online aspects of mobility, transport, cities, and objects. Based on this, Behrendt [131] developed the concept of smart velomobility, which is concerned with the networked practices, systems, and technologies of cycling. The author observes that the motor age, overshadowed by the digital age, entails not only motoring but also a speed change in the digital age. It brings together forms of cycling mobility (velomobility) with the smart/intelligent/code/data aspects of mobility [132]. The deployment of smart transport systems in cars and public transport is changing rapidly from a niche activity to a basic socio–technical mode, while intelligent cycling remains in a niche state and, therefore, has the potential for innovations in further integrating low carbon technology transitions [129]. Behrendt [131] hopes that both research and policy will increasingly use utopian approaches for the future of intellectual mobility, thereby overcoming the current divide between certain mobility options in the prevailing the internet of things and car-oriented visions for future mobility, rather than developing more radical approaches to unsustainable mobility, including focusing on active modes. 

#### 3.3.4. Green Tourism Transport Solution via Walkability

There is growing interest in making places greener by improving their walkability [132]. Schmeidler [133] observes that walking is a versatile mode of transport, and for many people, it is the only means of transportation. Moreover, as Wang and Wen [109] observe, there is an increasing emphasis on walking as an active form of sustainable mobility. Ram and Hall [116] state that walking is an essentially human activity. More recently, the authors in [116] observed that walking is a means for promoting greater health and well-being, community development, and more sustainable travel [134]. However, despite the importance of the topic of walking, there is currently no integrated treatment of the subject in the social science literature (see also Popp [135]). Hall and Ram [112] discuss walking as an important part of the tourist experience and a significant element of sustainable mobility. The authors [112] also emphasize the need to evaluate the tourist choices of hiking and transport options in order to encourage visitors to use active transport at targeted locations. Botella-Carrubi et al. [136] focused on pilgrimage travel. These trips are a great attraction for both pilgrims and tourists. The key elements in this type of travel are the travelers’ communality and their relationship with nature. Saunders et al. [119] described the results of a qualitative study on middle-aged adults who personally described an extensive long-distance walking experience. Miles [118] discussed the world’s first national inshore walkway and noted that the biggest advantage of this foot walk is that it is designed for both serious and casual participants, starting with a random stroll at a short distance followed by the more difficult hurdle of walking the whole route. UNWTO [134] noted that walking tourism was one of the most popular ways to experience a destination in 2019. 

Thus, possible solutions using green logistics for sustainable tourism include mobilities, bicycle tourism, the co-creation of smart velomobility, and walkability. Following Hoedʹs [114] notion that travel and tourism should no longer be isolated and understood separately from other forms of mobility (thus relating tourist activities, experiences, and destinations to (active) everyday mobility), the right to a clean environment could be ensured using these and other evolving solutions for a more sustainable future. 

The next section of this article discusses the challenges and importance of seeking balance to achieve the right to a clean environment by examining the context (the right to a clean environment), the challenge (climate change), and the solutions (green logistics for sustainable tourism).

## 4. Discussion: Seeking Balance for the Right to a Clean Environment

The integrative review highlighted the importance of seeking a balance between the right to a clean environment as the context, climate change as the challenge, and green logistics solutions for sustainable tourism as solutions. 

**Figure 1:**
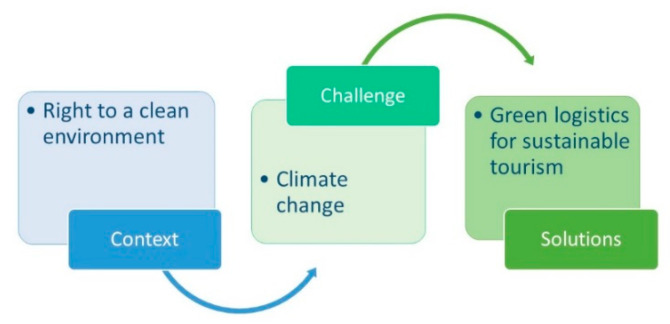
The “seeking balance” framework.

The concept of this discussion is outlined in Table 1. Accordingly, the discussion section is divided into the following sections: context, challenge, and solutions. The discussion is summarized by debating alternative travel solutions and is presented in the framework of “seeking balance” (Figure 1).

### 4.1. The Right to a Clean Environment as the Context

As previously noted, ensuring the human right to a clean, healthy, and safe natural environment and protecting the environment from negative impacts is one of the most pressing issues, from both socio–economic and legal–political perspectives. These issues depend on the recognition, regulation, and application of preventive environmental methods or measures seeking a harmonized legal framework at the national and international levels. Notably, in the current period of technical progress, the environment is affected by various negative factors, such as pollution, climate change, and nuclear dangers that threaten not only human life and health but also humanity as a whole. These negative outcomes are global issues and require both national and international unified responses and solutions. In the scientific literature and legal documents, the concept of a clean environment is not well established, and the most commonly used terms are the “right to a healthy and clean environment” and the “right to a healthy and safe environment”. According to Lewis [8], attempts to recognize the right to a good environment under international human rights law have few prospects of success. According to the author [8], the negative impact of the environment on human rights can be direct, because a polluted or damaged environment will directly affect a person’s access to his or her rights, or indirect, as poor environmental conditions could undermine the government’s ability to protect and enforce its citizens [8]. It should be noted that the current legislative proposals and concrete decisions to fulfill the right to a safe and clean environment have only distracted society from the important work that could be done to strengthen and clarify the relationship between human rights and the environment. 

### 4.2. Climate Change as the Challenge

Lewis [8,9] proposes that greater attention should be given to clarifying states’ obligations to respect, protect, and enforce human rights in the context of climate change and, in particular, how human rights laws can adapt to the international and long-term effects of climate change. For a more effective implementation of human rights for a clean and safe environment, legal practitioners, scientists, and public activists recommend that a human rights-based approach to climate change should focus on how existing rights can be enforced and implemented, rather than creating new laws or restrictions [3,8,9,10]. As Simpson et al. ([35], p. 12) point out, to adapt to climate change and reduce its contributions to the global emissions, it is essential that the tourism industry also make concerted efforts to reduce its environmental impact. In this difficult and problematic situation, the question remains: What should be done to at least partially resolve the issues discussed above? One of the solutions to reduce, or at least suspend, the impact of the aforementioned negative effects on the environment is to use the ideas of green logistics to achieve more sustainable tourism. 

### 4.3. Green Logistics for Sustainable Tourism as Solutions

As mentioned previously, tourism and transport pose some of the greatest threats to a clean and safe environment. The smart and targeted use of green logistics ideas in the tourism industry would help limit and reduce negative factors and at least partially achieve the right to a clean and safe environment. Behrendt [124] points out that the various benefits of cycling, such as better public health and well-being, lower emission values, affordability, increased physical activity, and reduced congestion and emissions, can be realized in the smart/digital world age. As delivery and passenger drones may soon come closer to real life implementation they will become more ‘tangible’ to wider parts of society, so as Kellermann et al. [113] point out, a true assessment of the environmental friendliness of drones therefore needs to include a stronger comparative perspective, taking into account other modes of transportation. As Wang and Wen [105] point out, creating a friendly environment for walking and cycling is essential to increase people’s daily physical activity levels and reduce car addiction. Nilsson [21] notices that in a city, there is a link between vehicles, vehicle speed, and social interaction, as areas dominated by walking or cycling provide more services and activities than car-dominated areas. Diaz-Soria [137] observes that tourists deliberately create distance from their destination, allowing them to enjoy the tourist experience as something exclusive. Nilsson [21] notes that the relationship between cycling, locals, and tourists is complex and requires further research. This is especially true in unconventional tourist areas, which cycling tourist flows can affect in unforeseen ways. Hoed [114] emphasizes the importance of continuous dialogue with slower and more age-inclusive attitudes. This is a fruitful quest for the future of tourism that will be sustainable for both people and places. The industry of tourism must adopt new ways of the thinking and, more importantly, act to develop strategies to make tourists greener and more socially respectful while they are traveling [133]. This would encourage tourists to start thinking about how they travel and to act according to their destinations. As Weston et al. [138] report, this would also support the strategies of sustainability in the infrastructure of sustainable transport and the development of sustainable growth in tourism. It would give tourists the opportunity to act in the same way that they think. It would let to solve challenges between locals, tourists and unique places, together looking for smart, more sustainable practices of green solutions.

### 4.4. The Framework “Seeking Balance”

Figure 1 shows the importance of seeking a balance between the context (the right to a clean environment), challenge (climate change), and solutions (green logistics for sustainable tourism). In this article, we did not seek to debate how to avoid or restrict an individual’s right to travel. Despite reducing tourist flows, this could also negatively impact the natural environment. We rather invite the reader to consider sustainable alternatives to travel that balance the right to travel with the right to a clean environment. These rights are clearly contradictory and influence each other. On the one hand, tourism can damage local communities through the intervention of outsiders in the target society. On the other hand, tourism can help preserve cultural and natural heritage. However, the paradox remains: The more people travel, the greater their threat to the quality of the environment. How can we reconcile and balance these rights? Are laws capable of solving this problem? It seems interesting to point out, moreover, that such challenging discussions started to emerge more in recent years. Gascón [139] argues that the right to tourist mobility can limit the mobility of locals, as well as their use and enjoyment of their resources (the right to tourism vs. the right to the city). Interestingly, transforming tourism into a right allows the debate to be observed. This debate, however, is no longer about whether the negative effects of tourism should be addressed through technological development and ourselves via regulatory measures (whose viability is questionable) or, conversely, through institutional intervention and austerity policies [139]. The debate instead focuses on how to manage conflicts of rights. As Gascón [139] observes, this conflict encompasses the rights of a citizen as a tourist versus the rights of a citizen as a resident and the right of tourists to travel around the planet versus the rights designed to ensure ecosystem resilience. The discussed sustainable tourism challenges related to the pro-environmental behavior of tourists and ecotourism also opens up a broader debate on issues of responsibility. Rossello et al. [140] discussed the widespread impact of natural disasters and unexpected events for all areas, including tourism, and highlighted the impact of disasters on tourist needs. Therefore, it seems particularly important (in this dispute on rights) to keep in mind the possible applications of correct technological (and other) solutions to this problem. Scott et al. [141] discuss the need for international tourism leadership to improve its sectoral scale, emissions, and monitoring capacity to meet the increasing requirements for transparency, as well as assess the risks of climate change and climate policy, foster greater collaboration to facilitate climate resilience, and accelerate technological, policy, and social innovation to put tourism firmly on a path toward a low-carbon economy. Also emphasized is the need for dialogue between tourism and the researchers of tourism.

Ultimately, the right to a clean environment (i.e., a safe, high quality, clean, sustainable, or good environment) is difficult to implement. The successful implementation of this right depends on the application of various complex ideas and rational solutions, such as tourism mobilities, bicycle tourism, the co-creation of smart velomobility, and walkability. The right to a clean environment could be ensured just carefully adapting these and other evolving solutions (e.g., various drone services) for a more sustainable future. 

Green logistics ideas and solutions that seek more sustainable tourism and related practices could help encourage the adoption of the discussed solutions aimed at reducing environmental impacts and, at the same time, contribute to the implementation of the personal right to a clean environment. Moreover, as Perkumienė and Pranskūnienė [142] note, the interdisciplinary discussions on overtourism have shown the importance of rethinking the concept of sustainability in tourism as a holistic principle of democracy and the degrowth movement. Thus, the same importance is also needed in rethinking the concept of sustainable logistics solutions in the tourism sector. Vidal-González and Sánchez [143] observe that the interest in and demand for an ‘authentic’, rural, primitive, natural, and immaterial heritage is booming in our postmodern society, which seeks to escape its harsh urban realities. This is a positive direction, as some of the presented solutions respond to the emerging reconsiderations of sustainability.

## 5. Conclusions

This integrative review invites the reader to consider the sustainable alternatives to travel that could balance the right to travel with the right to a clean environment, highlighting the importance of seeking a balance between the context (the right to a clean environment), challenge (climate change), and green solutions (green logistics for sustainable tourism).

The right to a clean environment should allow a person to live in a harmonious system, where environmental factors do not pose a risk to human health and well-being. The implementation of this right requires complex decisions. Lewis [8,9] proposes that greater attention should be given to clarifying states’ obligations to respect, protect, and enforce human rights in the context of climate change and, in particular, to understand how human rights laws can adapt to the international and long-term effects of climate change. Rethinking the possible green logistics solutions for sustainable tourism, such as tourism mobilities, bicycle tourism, the co-creation of smart velomobility, walkability, and others could help us also rethink how to balance, respect, protect, and enforce human rights in the present-day context of ongoing climate change challenges.

Optimistically, some of the presented solutions show how green logistics solutions in the tourism sector could be adopted for daily use and sustainable tourism experiences, seeking a more harmonious implementation of the individual rights to a clean environment. This integrative review paper is only preliminary work; further research is needed to discuss the evolving green solutions and challenges for more sustainable logistics and tourism and more effectively guarantee each citizen’s right to a clean and healthy environment.

## Figures and Tables

**Table 1:** The stages of the review.

No.	Stage of Review	Description of Review Stages
1.	Problem Identification	Theoretical and empirical discussions about the right to a clean environment considering green logistics and sustainable tourism
2.	Literature Search	We used open databases and open libraries to find articles about green logistics and tourism and the right to a clean environment: DOAJ (Directory of Open Access Journals): 239 articles on green logistics, five articles on green tourism/bicycle tourism, and 10 articles on logistics and cycling; IDEAS/RePEc: We found 52 results for “the right to a clean environment“, 419 results for “green logistics“, three results for “tourism and green logistics“; ScienceOpen: We found 113 results for „green logistics“, two results for “right to a clean environment“; SSRN eLibrary: We found 43 results for “green logistics“ and 88 results for “right to a clean environment“; WorldWideScience.org: We found 3173 results for “the right to a clean environment“; WorldWideScience.org: We found 1509 results for “green logistics solutions for tourism“; ScienceDirect: We found 640 results for “green logistics solutions for tourism“, ScienceDirect: We found 317 results for “green logistics and tourist mobility“, but only some of them concentrated on the right to a clean environment in a global warming context in logistics and tourism. No articles about green logistics solutions for tourism topic were found in the databases and the open libraries of IDEAS/RePEc, The OAPEN Library, OpenDOAR, Electronic Journals Library, WorldWideScience.org, EBSCO, and others. The literature was also searched for research on the right to a healthy, safe, and a clean environment, sustainable, green logistics, logistics and tourism). We then evaluated the data (theoretical and an empirical literature, the legal cases discussing the right to a clean environment, and green logistics and tourism).
3.	Data Evaluation	Theoretical and empirical literature, the legal cases discussing the right to a clean environment, and green logistics and sustainable tourism.
4.	Data Analysis	Theoretical and empirical literature and legal cases discussing the right to a clean environment, green logistics, and tourism were analyzed for the following topics: legal cases and conventions, literature on tourism, climate change, and sustainability, and literature on green logistics solutions for sustainable tourism.
5.	Presentation	The integrative review is presented in the results chapter (the integrated review data were organized under the following themes: the right to a clean environment and its legal regulations (Section 3.1); tourism challenges: considering climate change and sustainability (Section 3.2); green logistics for sustainable tourism (Section 3.3); the discussions chapter presents the conceptual framework (Figure 1) “Seeking the balance”.

**Table 2:** Legal documents related to a clean environment.

Year	Legal Cases and Conventions	Keywords
2015	Ashgar Leghari v. Federation of Pakistan. *No. 25501.* [66]	The right to a clean environment, fundamental rights, climate change, nuclear dangers, human life and health
1974	Nold v. KG v. Commission [69]	The right to a healthy, safe, and clean environment, the right to life, the right to respect the private and family life, and cases of pollution and noise
2004	ECHR case Öneryıldız v. Turkey [70]	The right to a healthy, safe, and a clean environment; the right to life, the right to respect private and family life, and freedom of expression
2015	Urgenda Foundation v. The State of the Netherlands, C/09/456689/HA ZA 13-1396 (24 June 2015) [71]	Greenhouse effects and emissions, particularly CO2
2005	Fadeyeva v. Russia [72]	Environmental factors, private life, the right to life, environmental contexts
2008	Budayeva and others v. Russia [73]	The right to life, the individual’s right to life as a result of the harmful effects of the environment; the right to respect private and a family life.
1976	X v. Iceland, 5 DR 86 [74]	Harmful effects of the environment; the right to respect private and family life.
2016	Kelsey Cascade Rose Juliana et al. v. United States of America [75]	
2009	Case Tatar v. Romania [76]	
2013	Case Kolyadenko v. Russia (2013) [77]	
1948	Universal Declaration of Human Rights [78]	The right to a clean and safe environment, fundamental rights, a healthy and clean environment.
1972	The Stockholm Declaration on the Human Environment [79]	The human right to a safe and clean environment; the global human impact on the environment and attempts to develop; solving the problems of preserving and improving a safe and clean human environment; decent living in an environment that can produce dignity and well-being
1992	Rio Declaration on Environment and Development [80]	The human right to a healthy and full life in harmony with nature; the rights of individuals to a safe environment, the right to a healthy and a productive life in harmony with nature.
1992	Treaty on European Union (TEU)/Maastricht Treaty [81]	The human right to a clean and a healthy environment; the protection of the environment.
1997	Kyoto Protocol to the United Nations Framework Convention on Climate Change [82]	Climate change; greenhouse effects by limiting CO2 emissions.
1997	Treaty of Amsterdam [83]	Endless development and stronger protection of the environment.
1998	Aarhus Convention [84]	A clean and healthy environment; the duty of states to ensure the rights of citizens to objective information, public participation in decision-making and environmental matters to ensure their right to live in an environment favorable to their health and well-being.

**Table 3:** Description of the literature on tourism, climate change, and sustainability.

Authors	Year	Journal/Book	Keywords
		Sustainability, Tourism and Climate	
Wang, Qiao, Cheng, Yanan Sun, He [28]	2019	International Journal of Low-Carbon Technologies	Acid rain pollution, concrete, damage, economic loss
Grimm, Alcântara, Sampaio [39]	2018	Brazilian journal of tourism research	Climate impacts, tourism, adaptation, mitigation
Muler, Coromina, Galí [87]	2018	Tourism Review	Sustainable tourism, social exchange theory, heritage towns
Mathew, Sreejesh [90]	2017	Journal of Hospitality and Tourism Management	Responsible tourism, sustainability, quality of life, destination management
Rogerson [95]	2016	Local Economy: The Journal of the Local Economy Policy Unit	Capacity building, climate change, local economic development, tourism
Mearns [38]	2016	African Journal of Hospitality, Tourism and Leisure	Climate change, tourism, global warming, sustainability, airlines
Tang [86]	2015	Tourism Management	Tourism, environment, relationship, coordination
Peeters, Eijgelaar [97]	2014	Tourism Management	Climate change, sustainable development, air transport volume, mitigation of emissions
Hall, Scott, Gössling [47]	2013	Sustainable Development	Green growth, aviation, greenhouse gas emissions, tourism policy
Scott, Hall, Gössling [37]	2012	Tourism and Climate Change: Impacts, Adaptation and Mitigation	Tourism, climate change
Cavagnaro, Curiel [94]	2012	The Three Levels of Sustainability	Sustainable tourism, clean environment
Becken, Hay [96]	2012	Climate Change and Tourism: From Policy to Practice	Climate change, tourism, policy, practice
Page [15]	2011	Tourism Management: An Introduction.	Tourism, sustainability, tourism management, pro poor tourism and poverty
Cohen, Higham [98]	2011	Current Issues inTourism	Changing consumer tourism behavior, climate change, social norms
Simpson, Gössling, Scott, Hall, Gladin [35]	2008	Climate Change Adaptation and Mitigation in the Tourism Sector: Frameworks, Tools and Practices	Climate Change, adaptation, tourism, tools
Scheyvens [99]	2007	Current Issues in Tourism	Poverty, development, pro-poor tourism

**Table 4:** Description of literature sources on green solutions for tourism.

Authors	Year	Journal/Book	Keywords
Kim, Hwang [55]	2020	Journal of Hospitality and Tourism Management	Pro-environmental role, drone food delivery services, norm activation model, theory of planned behavior, product knowledge
Kellermann, Biehle, Fischer [113]	2020	Transportation Research Interdisciplinary Perspectives	Environment, drones, logistics, passenger transportation
Hwang, Kim [57]	2019	Business Strategy and the Environment	Environment, drone food delivery services, green image
Nilsson [21]	2019	Journal of Tourism Futures	Cycling, bicycle tourism, urban tourism, mobility culture
Hoed [114]	2019	Journal of sustainable tourism	Tourism, active mobility, cycling, well-being
Seroka-Stolka, Ociepa-Kubicka [48]	2019	Transportation Research Procedia	Green logistics, circular economy, green practices
Smith, Robbins, Dickinson [112]	2019	Journal of Sustainable Tourism	Sustainable travel, tourism, social practices
Hanna, Adams [59]	2019	Journal of Sustainable Tourism	Sustainable transport, sustainable barriers, transport futures
Kantenbacher, Hanna, Miller, Scarles, Yang [115]	2019	Journal of Sustainable Tourism	Environment, air travel, tourist behavior, climate change
Scuttari, Orsi, Bassani [116]	2019	Journal of Sustainable Tourism	Sustainable tourism, tourism transport, alternative transportation, behavioral change
Hall, Ram [117]	2019	Journal of Sustainable Tourism	Active transport, transport choice, built environment, walkability, accessibility
Miles [118]	2018	The Routledge International Handbook of Walking	Transport, walking
Saunders, Weiler, Laing [119]	2018	The Routledge International Handbook of Walking	Walking experiences
Gavrilović, Maksimović [52]	2018	Strategic management	Green logistics initiatives, green management, environment, sustainable tourism
Peeters, Gössling, Klijs, Milano, Novelli, Dijkmans, Eijgelaar, Hartman, Heslinga, Isaac, Mitas, Moretti, Nawijn, Papp, Postma [120]	2018	Research for TRAN ^1^ Committee	Overtourism, EU, Tourism transportation, sustainable tourism
Ram, Hall [121]	2018	International Journal of Tourism Cities	Walkability, cities, destinations, accessibility, tourism walking
Broman, Robèrt [110]	2017	Journal of Cleaner Production	Strategic sustainable development, sustainability principles, sustainability science
Cass, Faulconbridge [122]	2017	Mobilities	Mobility, travel, practice, affect, transport mode changes, lower carbon mobility
Berrada [123]	2017	Journal of International Business Research and Marketing	Value co-creation, tourism, tourist experience, sustainability, new technologies, internet
Wang, Wen [109]	2017	Urban Science	Sustainable mobility, active transportation, built environment, cycling, walking
Karia, Asaari [108]	2016	Proceedings of the 2016 International Conference on Industrial Engineering and Operations Management	Green logistics practice, environmental performance, economic performance, quality of life, third-party logistics, sustainable development, carbon emission
Behrendt [124]	2016	Journal of Transport Geography	Smart Cities, Cycling, Sustainable Transport, Mobility, Intelligent Transport, Internet of Things
Lee [125]	2016	Social and Cultural Geography	Cycling, bicycle commuters, mobility, embodiment, bike to work.
Zhang, Zhang, Duan, Bryde [111]	2015	Journal of Cleaner Production	Green practices, transportation, bike-sharing, sustainable development, sustainability
McKinnon [105]	2015	Green Logistics: Improving the Environmental Sustainability of Logistics	Environmental sustainability, green practices, regulations on emission level, public policy interventions
Rodriguez, Slack, Comtois [126]	2013	Green Supply Chain Management	Green activities, green transportation, sustainable urban mobility

^1^ TRAN = Committee on Transport and Tourism.
